# Prediction of long-term kinetics of vaccine-elicited neutralizing antibody and time-varying vaccine-specific efficacy against the SARS-CoV-2 Delta variant by clinical endpoint

**DOI:** 10.1186/s12916-022-02249-9

**Published:** 2022-01-28

**Authors:** Xinhua Chen, Wei Wang, Xinghui Chen, Qianhui Wu, Ruijia Sun, Shijia Ge, Nan Zheng, Wanying Lu, Juan Yang, Lance Rodewald, Hongjie Yu

**Affiliations:** 1 School of Public Health, Fudan University, Key Laboratory of Public Health Safety, Ministry of Education, Shanghai, 200032 China; 2 National Immunization Programme, Chinese Center for Disease Control and Prevention, Beijing, China

**Keywords:** COVID-19 vaccines, SARS-CoV-2 Delta variants, Time-varying efficacy, Prediction

## Abstract

**Background:**

Evidence on vaccine-specific protection over time, in particular against the Delta variant, and protection afforded by a homologous third dose is urgently needed.

**Methods:**

We used a previously published model and neutralization data for five vaccines—mRNA-1273, BNT162b2, NVX-CoV2373, V01, and CoronaVac— to evaluate long-term neutralizing antibody dynamics and predict time-varying efficacy against the Delta variant by specific vaccine, age group, and clinical severity.

**Results:**

We found that homologous third-dose vaccination produces higher neutralization titers compared with titers observed following primary-series vaccination for all vaccines studied. We estimate the efficacy of mRNA-1273 and BNT162b2 against Delta variant infection to be 63.5% (95% CI: 51.4–67.3%) and 78.4% (95% CI: 72.2–83.5%), respectively, 14–30 days after the second dose, and that efficacy decreases to 36.0% (95% CI: 24.1–58.0%) and 38.5% (95% CI: 28.7–49.1%) 6–8 months later. Fourteen to 30 days after administration of homologous third doses, efficacy against the Delta variant would be 97.0% (95% CI: 96.4–98.5%) and 97.2% (95.7–98.1%). All five vaccines are predicted to provide good protection against severe illness from the Delta variant after both primary and homologous third dose vaccination.

**Conclusions:**

Timely administration of third doses of SARS-CoV-2-prototype-based vaccines can provide protection against the Delta variant, with better performance from mRNA vaccines than from protein and inactivated vaccines. Irrespective of vaccine technology, a homologous third dose for all types of vaccines included in the study will effectively prevent symptomatic and severe COVID-19 caused by the Delta variant. Long-term monitoring and surveillance of antibody dynamics and vaccine protection, as well as further validation of neutralizing antibody levels or other markers that can serve as correlates of protection against SARS-CoV-2 and its variants, are needed to inform COVID-19 pandemic responses.

**Supplementary Information:**

The online version contains supplementary material available at 10.1186/s12916-022-02249-9.

## Background

The ongoing coronavirus disease 2019 (COVID-19) global pandemic, caused by severe acute respiratory syndrome coronavirus 2 (SARS-CoV-2), has led to immense mortality and morbidity and huge socioeconomic damage [1]. Safe, effective, and deployable vaccines are useful tools to control virus transmission, build population immunity, and help bring the world back to pre-pandemic normalcy [2]. However, protection provided by currently available COVID-19 vaccines is becoming compromised over time due to waning immunity after vaccination and prominence of newly emerged SARS-CoV-2 variants.

Although the observed dynamics of vaccine-elicited neutralizing antibody—a highly predictive bio-marker of humoral immunity—have been well-characterized, few prospective studies are available on long-term kinetics of vaccine-induced neutralizing antibody or protective efficacy/effectiveness. Compared to neutralizing antibody levels induced by natural infection, vaccination may induce similar or lower neutralizing antibody levels [3] that decay faster [4]. As of this writing, several studies have reported 6–8-month antibody kinetics after primary two-dose vaccination with three WHO emergency-use-listed vaccines: BNT162b2 [5], mRNA-1273 [6], and Ad26.COV2.S [7]. However, for most COVID-19 vaccines, long-term neutralizing antibody kinetics after homologous booster doses and resulting vaccine effectiveness over time against the prototype strain and the Delta variant are still unknown. The extent to which a homologous booster dose provides protection above and beyond primary series vaccination is important but not known. Generating such evidence requires labor-intensive, time-consuming, long-term study. A recently published modeling study that included booster vaccination predicted vaccine effectiveness over time for different clinical outcomes. However, the study did not evaluate effectiveness by vaccine platform and age-group, nor did it include effectiveness against infection as an outcome [8].

Duration of protection of primary vaccination and the relationship between protection and vaccine-elicited neutralizing antibody levels are not yet fully characterized. There is limited evidence on the timing of a homologous third dose relative to primary vaccination and duration of protection. In our systematic review, we summarize kinetics of vaccine-induced neutralizing antibody 5–8 months after primary vaccination and 1 month after a homologous third dose for two mRNA vaccines (BNT162b2 and mRNA-1273), two protein subunit vaccines (NVX-CoV2373 and V01), and one inactivated vaccine (CoronaVac). Using a previously verified model that correlates neutralizing antibody levels and vaccine protection [8, 9], we predict age-specific vaccine efficacy against the Delta variant over time, across different vaccines, and against three clinical endpoints—infection, symptomatic COVID-19, and severe COVID-19.

## Methods

### Data sources

#### Individual-level neutralizing antibody titer data for CoronaVac

Randomized, double-blind, placebo-controlled, phase 1/2 clinical trials of CoronaVac in healthy adults aged 18 years and older were performed in Jiangsu province and Hebei province, China, as previously detailed [10, 11]. To evaluate persistence of CoronaVac vaccine-induced neutralizing antibody titers, blood specimens were obtained on days 0, 28, and 56 after the first dose; 6 months after two-doses; and days 7 and 14 or 28 after a third dose (Additional file 1, Fig. S1). Neutralizing antibody titers to infective SARS-CoV-2 (virus strain SARS-CoV-2/human/CHN/CN1/2020, GenBank number MT407649.1) were quantified using a micro cytopathogenic effect assay, previously described [10, 12].

#### Neutralizing antibody dynamics with different vaccines with time-specific aggregated data collected from a comprehensive literature search

Using predefined search terms, we conducted a search for studies that reported dynamics of neutralizing antibodies and impact of a homologous booster dose among vaccine recipients in five databases—three peer-reviewed databases (PubMed, Embase, and Web of Science) and two preprint servers (medRxiv, bioRxiv) (Additional file 1, Table S4). We obtained official reports from vaccine companies to supplement retrieved data. Inclusion and exclusion criteria, as well as flowchart of selecting eligible studies are shown in the Additional file 1 (Table S5, Fig. S7). From the eligible studies, we abstracted vaccine name, vaccine developer, numbers and ages of study participants, vaccination schedule, type of neutralization assay (live or pseudo virus), and geometric mean titers (GMTs) over time after vaccination (Additional file 1, Table S6).

#### In vitro cross neutralization titers against prototype and Delta variant strains

We conducted a systematic search to update a previously reported meta-analysis of in vitro neutralization titers in individuals who have been vaccinated with prototype-strain-based vaccines against both SARS-CoV-2 prototype strains and variants [3]. We calculated n-fold-reductions of neutralizing antibodies against the Delta variant compared to the prototype strain for different types of neutralization assays (Additional file 1, Table S8).

### Statistical analysis

#### Neutralizing antibody dynamics fitted by a GAM model with individual data for CoronaVac

Titers of CoronaVac vaccine-induced SARS-CoV-2 neutralizing antibody were log-transformed before statistical analyses. Different from our previously qualitative analyses of CoronaVac vaccine-induced antibody titer data [13], we fit the kinetics of SARS-CoV-2 neutralizing antibody after vaccination using a generalized additive model (GAM) that allows for flexible specification of dependence of response to covariates. For modeling dynamics of SARS-CoV-2 neutralizing antibody, we assumed a GAM model with Gaussian distribution, which is commonly used to model antibody titers that involve evaluation of vaccine efficacy. Comparisons between models were made based on Bayesian information criteria (BIC) and Akaike information criteria (AIC). Given curve-fitting uncertainties of antibody titer kinetics in the 120–209 days after the first-dose of CoronaVac due to a paucity of observed immunogenicity data, we used multivariate imputation methods to impute immunogenicity data using immunogenicity data from participants who received other dosing schedules. We obtained imputed data with state-of-the-art multivariate imputation by the pan algorithm, which employs Markov Chain Monte Carlo (MCMC) techniques to draw replacements for multilevel missing values in databases [14]. As did Khoury and colleagues [9], we used a protective threshold of 33 for CoronaVac vaccine, which was defined as a neutralization titer at which CoronaVac will have 50% protective efficacy.

#### Predicting vaccine efficacy over time across different vaccines and clinical endpoints

Khoury and colleagues established a readily extrapolated model framework to predict vaccine efficacy against both SARA-CoV-2 prototype strain and variants [8, 9]. We used this model to predict vaccine protection against prototype strain based on the relationship between neutralizing antibody level and vaccine efficacy (Eq. 1); we used an integral based on normal distribution of neutralization level to calculate the probability of being protected (equation 2) [9].

1 $$ {E}_I\ \left(n|{n}_{50},k\right)=\frac{1}{1+{e}^{-k\left(n-{n}_{50}\right)}}, $$

2 $$ P\ \left({n}_{50},k,{\mu}_{\mathrm{s}},{\sigma}_{\mathrm{s}}\right)={\int}_{-\infty}^{+\infty }{\mathrm{E}}_I\ \left(n|{n}_{50},k\right)\ f\left(n|{\mu}_{\mathrm{s}},{\sigma}_{\mathrm{s}}\right) dn, $$

We used a logistic model to fit Eq. (1), where *E*_*I*_ is the vaccine efficacy given the log-transformed neutralizing antibody titer n, and *n*_*50*_ is the neutralization titer at which an individual will have a 50% protective efficacy. The parameter *k* controls the steepness of the logistic function. The relationship for different clinical endpoints was developed by changing *n*_*50*_ and *k.* For Eq. (2), assuming that neutralizing antibodies follow a normal distribution with mean *μ*_*s*_ and standard deviation *σ*_*s*_, *f* indicates the probability density function of neutralization titer, and *P* represents the proportion of vaccinated population in study *s* that will be protected. To enhance comparability between different studies with different neutralization assays, the neutralization titer (*μ*_*s*_) was normalized with means of titers against prototype strains in corresponding convalescent individuals reported in phase I/II trials. Confidence intervals of predicted efficacy against the prototype strain were estimated by using the Hessian H and standard error (s.e. = sqrt(diag(H-1))); 95% CIs were calculated as ± 1.96*s.e. of the estimated parameters [9].

For predictions of vaccine efficacy against the Delta variant, we added log-transformed n-fold-reductions of the Delta variant on neutralizing antibody levels into Eq. (3) and predicted variant-specific efficacy by using Eqs. (1) and (2) [15].

3 $$ {\mu}_s^v={\mu}_s+{\overline{F}}^v, $$

where *F*^*v*^ is the mean log-transformed n-fold-change (vaccine-specific) in neutralization titer against the Delta variant, *μ*_*s*_ is the normalized neutralization titer (vaccine-specific) against the prototype strain, and *μv s* is the normalized neutralization titer (vaccine-specific) for the Delta variant. Confidence intervals of predicted efficacy against the Delta variant were calculated by imputing the 95% confidence intervals of n-fold changes of neutralization titers.

## Results

### Neutralizing antibody dynamics from different vaccines

**Fig. 1:**
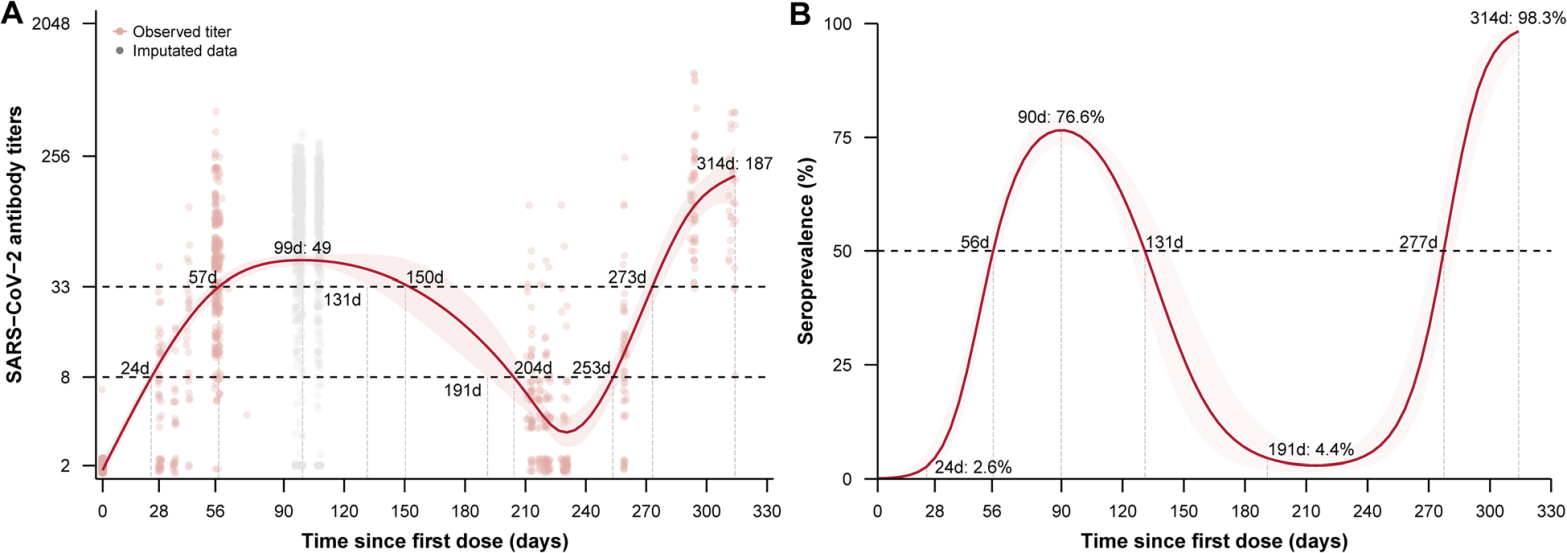
CoronaVac vaccine-induced SARS-CoV-2 neutralizing antibody response and corresponding seroprevalences after two primary doses plus a third dose of CoronaVac vaccine. **A** Fitted SARS-CoV-2 antibody titer dynamics. **B** Fitted seroprevalence, using a protective threshold of 33

For CoronaVac vaccine, immunogenicity data are from a phase 1/2 clinical trial in 244 healthy adults as described in the “Methods” section and detailed in Additional file 1, Table S1. The time to reach a protective threshold was 26–31 days after the second dose of CoronaVac (Additional file 1, Fig. S3). A monotonic increase was observed until two months after the second dose of CoronaVac vaccine, with a peak neutralizing antibody titer of 49 (95% CI: 46–54) and a seroprevalence of 76% (Fig. 1). The model estimated that individuals lost immunity 3.4 to 4.0 months after the primary two-dose series (Fig. 1). Participant age had little effect on the kinetics of CoronaVac vaccine-induced SARS-CoV-2 antibody (Additional file 1, Fig. S3), and the half-life of CoronaVac vaccine-induced SARS-CoV-2 antibody was estimated to be 57 days in all participants (Additional file 1, Fig. S5). Assuming 2.4-fold (95% CI: 1.1–5.2) reductions of antibody levels against the Delta variant compared to prototype strain, the time to the maximum titer was 2.3 months after completion of the second dose (Additional file 1, Fig. S6), a finding that was consistent between younger adults and older adults. In participants vaccinated with two primary doses plus one homologous booster dose, the time to the maximum concentration of 187 (95% CI: 120–293) was observed 1 month after homologous third doses. Compared with participants vaccinated with two primary doses, a 3.8-fold change in maximum concentration was observed by addition of the homologous third dose (Fig. 1). The percent of participants with protective antibody titers increased to a peak of 98.3% 28 to 30 days after a homologous third dose.

**Fig. 2:**
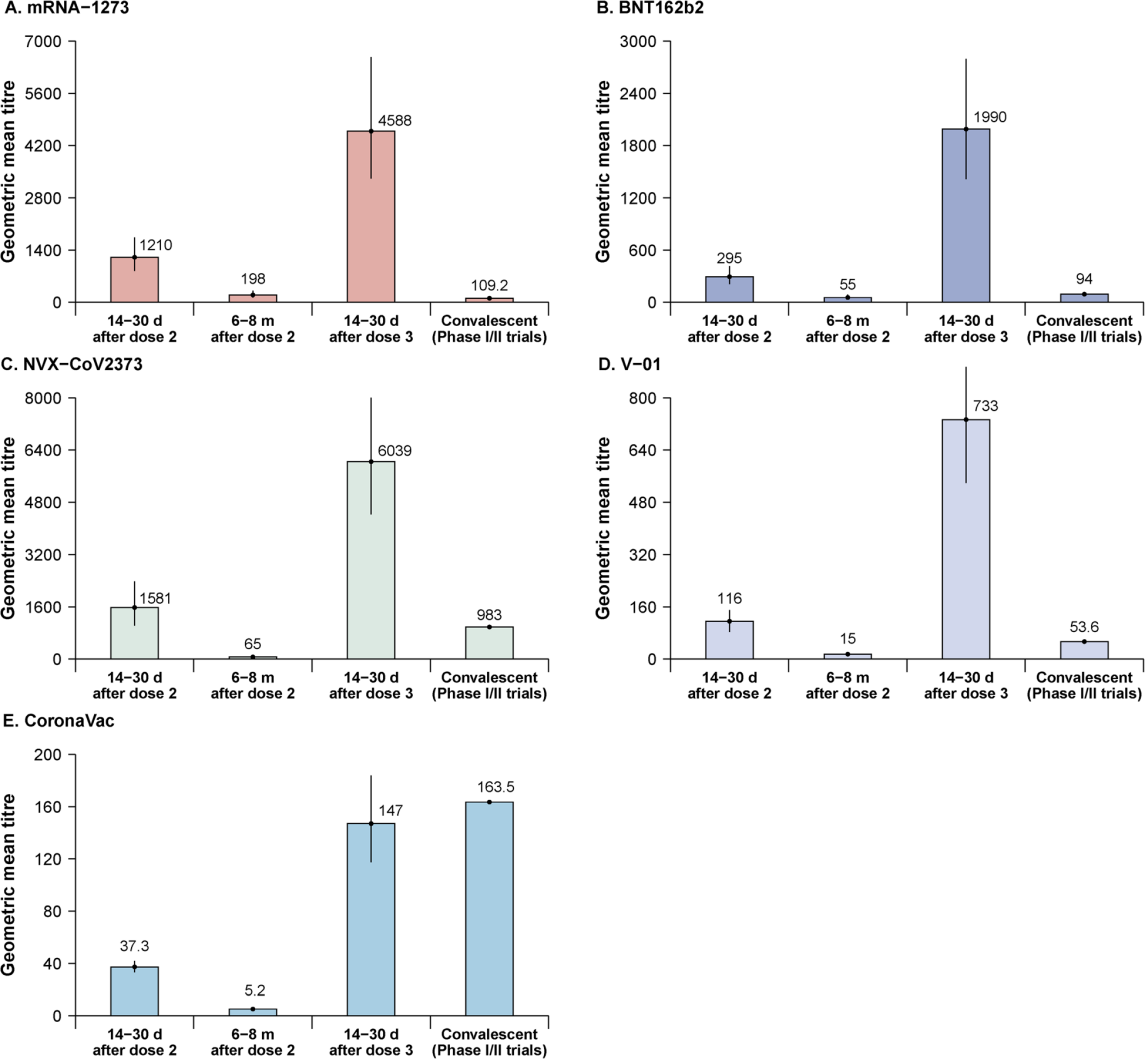
Vaccine-induced SARS-CoV-2 neutralizing antibody response by time period and natural-infection-induced neutralizing antibody response for convalescent patients in clinical trials. **A** mRNA-1273, **B** BNT162b2, **C** NVX-CoV2373, **D** V01, and **E** CoronaVac. The number on the top of the bar represents the GMT, and the vertical line represents the 95% confidence interval. The confidence interval of neutralizing antibody level for NVX-CoV2373 and V01 were digitally extracted from pictures. The neutralization assay for mRNA-1273 was a pseudovirus neutralization assay, while live virus neutralization assays were used for the other vaccines

In our literature search, we identified four studies that reported neutralizing antibody dynamics and antibody levels after a homologous third dose. The studies were of BNT162b2, mRNA-1273, NVX-CoV2373, and V01 vaccines—all two-dose primary-series vaccines with 21–28-day intervals between doses and studied with a homologous booster dose administered 5–8 months after the second dose. Neutralizing antibodies increased after primary series vaccination and reached peaks in one month, declining to relative low levels after 5–8 months. Booster vaccination induced higher levels of neutralizing titers compared with titers induced by two primary doses. Using plaque reduction neutralizing tests, the reported neutralization titer induced by BNT162b2 for individuals aged 18–55 years was 497 (95% CI: 248–979) on day 7 after the second dose, then declined to 387 (95% CI: 245–591) 1 month after the second dose, and to 83 (95% CI: 35–194) 8 months after the second dose. After a homologous third dose, the titer increased to 1754 (95% CI: 1260–2504) on day 7 and to 2119 (95% CI: 1257–3393) 1 month after the third dose (Additional file 1, Table S6). The immunogenicity pattern among elderly persons (65–85 years) was similar to that of younger adults, but with lower levels of neutralizing antibodies. For mRNA-1273, neutralization titers assessed with a pseudovirus neutralization assay were 1210 (95% CI: 840–1740) 1 month after the second dose, then decreased to 198 (95% CI: 126–315) 6–8 months after the second dose, and increased to 4588 (95% CI: 3315–6572) 14–30 days after a homologous third dose (Fig. 2). For NVX-CoV2373, the live-virus neutralization titer was 1581 (95% CI: 1030–2379) 2 weeks after the second dose, declining to 65 (95%CI: 41–99) 6 months after the second dose, and increasing to 6039 (95% CI: 4433–7998) 1 month after a homologous third dose (Fig. 2). For V01, the live-virus neutralization titer was 116 (95% CI: 83–150) 2 weeks after the second dose, declining to 15 (95% CI: 11–19) 5 months after the second dose, and increasing to 733 (95% CI: 539–895) 1 month after a homologous third dose (Fig. 2).

### Prediction of efficacy across different vaccines and clinical endpoints for prototype and Delta

**Fig. 3:**
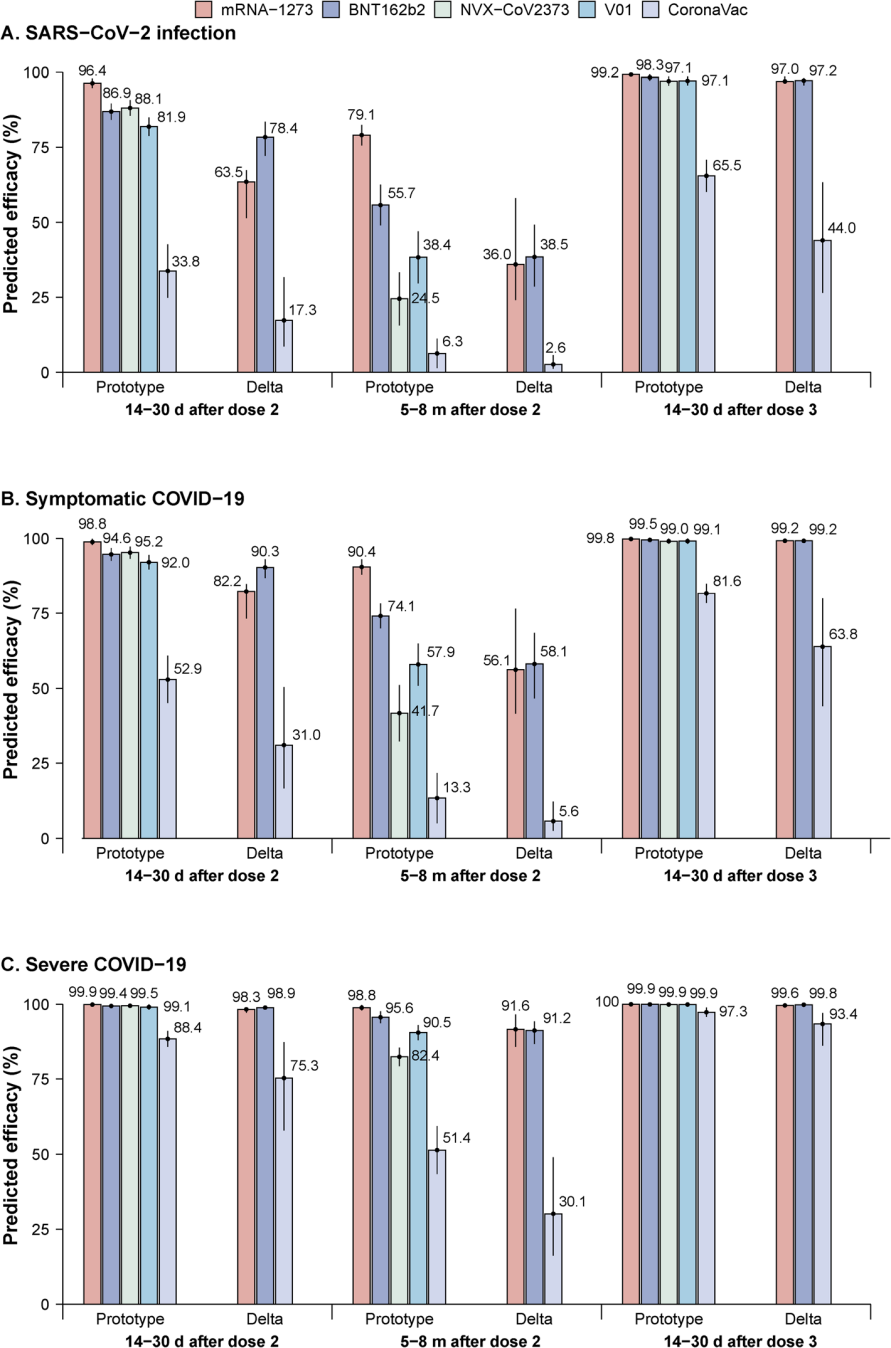
Predicted time-varying efficacy against both prototype strains and the Delta variant across three clinical endpoints. **A** SARS-CoV-2 infection, **B** symptomatic COVID-19, and **C** severe COVID-19. The number on the top of the bar represents the predicted efficacy, and the vertical line represents the 95% confidence interval

Predicted efficacy levels were consistent with changes in neutralizing titers over time and across different clinical endpoints. Predicted efficacy decreased starting from 14 to 30 days after primary series vaccination to lower values approximately 5–8 months after the second dose. Homologous third-dose vaccination increased protection from both the prototype strain and the Delta variant. For vaccine protection against infection caused by the Delta variant, predicted efficacy levels of the two mRNA-based vaccines, mRNA-1273 and BNT162b2, were 63.5% (95%CI: 51.4–67.3%) and 78.4% (95% CI: 72.2–83.5%), respectively, approximately 14–30 days after the second dose. Efficacy levels subsequently decreased to 36.0% (95% CI: 24.1–58.0%) and 38.5% (95% CI: 28.7–49.1%) 5–8 months after the second dose. Estimated efficacy levels against the Delta variant were 97.0% (95% CI: 96.4–98.5%) and 97.2% (95.7–98.1%) 14–30 days after a homologous third dose. Decreases in efficacy over time against the prototype strain were less than decreases in efficacy against the Delta variant, with an average protection of 91.7% (arithmetic mean) 30 days after dose 2, 67.4% 6–8 months after dose 2, and nearly 99% 14–30 days after a homologous third dose for either of the mRNA vaccines. These predicted efficacy levels are higher than efficacy predicted against the prototype strain induced by vaccination with NVX-CoV2373 and V01 in a corresponding time period (Fig. 3A).

For protection from symptomatic illness, the two mRNA vaccines provide good protection against both prototype and Delta strains, with over 50% protection sustained through 5–8 months after the second dose. The two mRNA vaccines can provide similar protection against the Delta variant, with 82.2% (95% CI: 73.3–84.7%) and 90.3% (95% CI: 86.7–92.9%) protection 14–30 days after the second dose, 56.1% (95% CI: 41.5–76.5%) and 58.1% (95% CI: 46.7–68.5%) 5–8 months after the second dose, and approximately 100% 14–30 days after a homologous third dose. Predicted efficacy of CoronaVac against the Delta variant was lower than efficacy predicted for the mRNA vaccines after two doses. Predicted efficacy increased to 63.8% (95% CI: 44.0–80.0%) 14–30 days after the third dose (Fig. 3B).

For severe COVID-19, predicted efficacy levels against the Delta variant for mRNA-1273, BNT162b2, and CoronaVac were 98.3% (95% CI: 97.1–98.6%), 98.9% (95% CI: 98.4–99.2%), and 75.3% (95% CI: 57.9–87.3%) between 14 and 30 days post dose 2; 91.6% (95% CI: 85.8–96.6%), 91.2% (95% CI: 86.8–94.3%), and 30.1% (95% CI: 16.2–49.0%) 6–8 months post dose 2; and 99.6% (95% CI: 99.5–99.8%), 99.8% (99.7–99.9%), and 93.4% (86.2–97.0%) 14–30 days after the homologous third dose (Fig. 3C).

### Prediction of age-specific efficacy across different vaccines and clinical endpoints for prototype and Delta

**Fig. 4:**
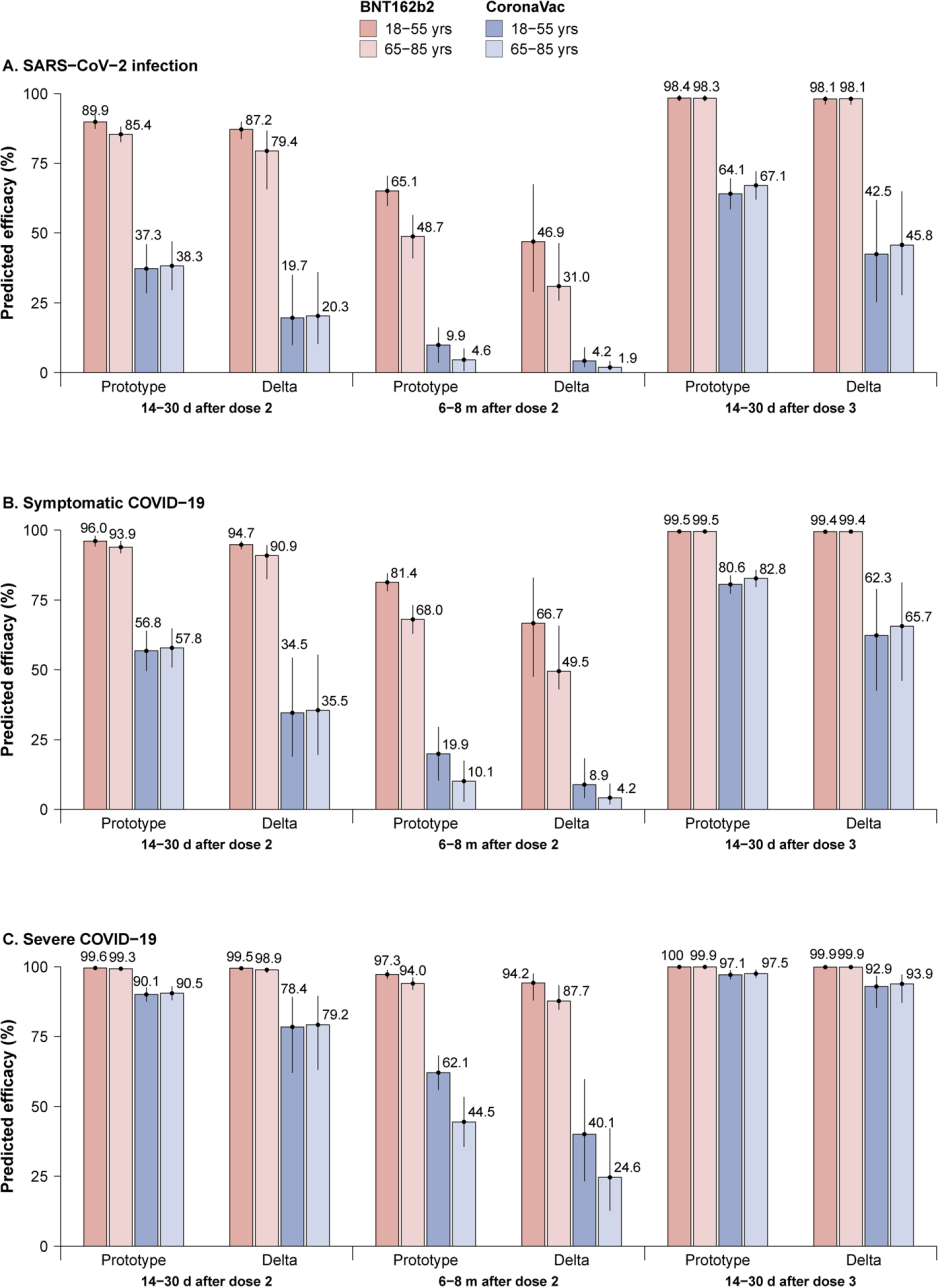
Predicted age-specific time-varying efficacy against both prototype strains and the Delta variant across three clinical endpoints. **A** SARS-CoV-2 infection, **B** symptomatic COVID-19, and **C** severe COVID-19. The number on the top of the bar represents the predicted efficacy, and the vertical line represents the 95% confidence interval

Age-specific efficacy was estimated for BNT162b2 and CoronaVac with younger (18–55 years) and older (65–85 years) age groups. Predicted protective efficacy was higher for BNT162b2 than for CoronaVac over time against both prototype and Delta variant strains and across different clinical endpoints and different age groups. For adults aged 18–55 years, predicted efficacy levels against symptomatic illness from the Delta variant for BNT162b2 were 94.7% (95% CI: 93.1–96.0%) 14–30 days after the second dose, 66.7% (95% CI: 47.5–83.0%) 6–8 months after the second dose, and 99.4% (95% CI: 98.7–99.7%) 14–30 days after the third dose. Corresponding values for CoronaVac against the Delta variant were 34.5% (95% CI: 18.9–54.3%), 8.9% (95% CI: 4.0–18.3%), and 62.3% (95% CI: 42.5–79.0%). Efficacy levels were lower for older adults than for younger adults, with 90.9% (95% CI: 82.5–94.5%) predicted protective efficacy 14–30 days after dose 2, 49.5% (95% CI: 43.0–65.8%) 6–8 months after dose 2, and 99.4% (95% CI: 98.8–99.7%) 1 month after the third dose for older adults vaccinated with BNT162b2 (Fig. 4B). For the other two clinical endpoints, predicted efficacy levels were slightly higher for younger adults than for older adults against both prototype and Delta variant strains (Fig. 4A, C).

## Discussion

Our study predicted long-term kinetics of vaccine-induced neutralizing antibodies for four COVID-19 vaccines in younger and older adults. We then predicted vaccine efficacy over time against prototype and Delta variant strains by these vaccines using three clinical endpoints in both age groups. We found similar patterns of vaccine-induced neutralizing antibodies over time, with booster vaccination producing the highest levels of neutralization titers compared with primary series vaccination-induced titers. We found that predicted efficacy declined from 14 to 30 days after the last primary series dose to low levels 5–8 months later and that homologous third doses, given approximately 6 months after the primary series will provide greater protection than the primary series against both prototype and Delta variant strains. Consistent with previous studies, we found that predicted efficacy levels of primary series and homologous third doses for the two mRNA vaccines were better than predicted efficacy levels from subunit protein vaccines, NVX-CoV2373 and V01, and from an inactivated vaccine, CoronaVac. However, we predict that all vaccines can provide good protection from the Delta variant against severe illness shortly after the primary series and after homologous third dose, suggesting that timely booster vaccination of any of these prototype-based vaccines can provide protection from Delta variant illness.

Several studies have shown that vaccine-induce neutralizing antibody responses are a highly predictive proxy for vaccine protection [9, 15–17]. By correlating vaccine efficacy and level of neutralizing antibodies, protective effect over time can be estimated based on time-varying neutralization titers. Patterns of predicted efficacy over time are highly consistent with the kinetics of neutralizing antibody induced by vaccine administration [18–21]. Doria-Rose and colleagues found that neutralizing antibodies elicited by two doses of mRNA-1273 could persist 6 months, and Barouch and colleagues reported that Ad26.COV2.S vaccine, a non-replicating adenovirus vectored vaccine, elicits humoral responses of at least 8 months duration after vaccination [6, 7]. A real-world observational study showed that mean levels of neutralizing antibodies decline to the lower limit of seropositivity 151–332 days after a second dose of inactivated COVID-19 vaccines [22]. These results are supported by our study, in that vaccine protection decreased after primary series vaccination and that the protection level for some vaccines 5–8 months later was less than 50%—especially for protection against variants. Our study also found that a homologous third dose can provide better vaccine efficacy for three clinical endpoints, a finding that is consistent with several studies showing that booster doses given 6 months after primary series vaccination increase neutralizing antibodies to higher levels than primary vaccination [13, 23]. Immunogenicity of a third dose, boosting previously established immunity from natural infection or vaccination as potential mechanisms of cross-protection, needs further study.

Compared to the dynamics of neutralizing antibodies in convalescent patients, a cohort study found that 90% of participants still had neutralization titers of ≥ 1:20 6–8 months post symptom onset and that even low levels of neutralizing antibody (1:20) are associated with a substantial degree of protection against COVID-19 in nonhuman primates [24, 25]. Although correlates of protection acquired from natural infection have not yet been established for COVID-19, and correlates of protection from illness of various severity levels remain unknown, neutralizing antibodies induced by virus infections may serve as a proxy to indicate protection. However, it should be noted that neutralizing antibodies are not the sole mechanism of protection from COVID-19; more research on the protection provided by cellular immunity is needed.

Our study showed that protective levels for different vaccines decreased by varying degrees 5 to 8 months after primary series vaccination. Declines for a subunit protein vaccine and an inactivated vaccine were more pronounced compared with the two mRNA vaccines—especially against the Delta variant. It has been widely accepted that immunogenicity against prototype SARS-CoV-2 from mRNA vaccines is better than immunogenicity from other vaccines made with other technological platforms [26–28]. This is consistent with our finding that the decline of neutralizing antibodies was smaller and slower with mRNA vaccines than for protein and inactivated vaccines. Additionally, in vitro cross neutralization assays showed that reduction of neutralizing antibodies against the Delta variant is significantly less than for vaccines made with other platforms and for convalescent sera [3]. These factors likely contribute to the relatively higher and sustained predicted efficacy for mRNA vaccines. Boosting induces higher antibody responses to the prototype strain than does primary series vaccination, and the high magnitude of the response may provide protection against the Delta variant with different clinical severities. Maximizing neutralizing antibody response to the prototype strain, in the absence of Delta-variant-specific vaccines, may be an effective strategy to prevent or control Delta variant outbreaks.

Our study estimated vaccine-specific protection over time and the impact of giving homologous third doses. Our findings provide evidence for public health decision-makers on the timing of booster doses and preparation for outbreaks. We estimated that the efficacy of BNT162b2 against the prototype strain 1 month to 5 to 8months after the primary series are 94.6% (92.5–96.8%) and 74.1% (69.9–78.2%), respectively. These results are similar to a clinical trial that reported that efficacy peaked at 96.2% (93.3–98.1%) within 2 months and declined to 83.7 (95% CI: 74.7–89.9%) 4 months after the second dose, for an average decline of 6% every 2 months [29]. Assuming constant rate of decline, efficacy on month 6 after the second dose will be 77.7%, which is slightly higher than our predicted estimate of 74.1% [30]. Our study predicted that efficacy of mRNA-1273 is 90.4% (95% CI: 87.9–93.0%) 6 to 8 months after the second dose, which is comparable to the reported efficacy (92.4%, 95% CI: 84.3–96.8%). Our study predicted age-specific vaccine protection by different time periods after two-dose primary series, showing that efficacy for younger adults is slightly higher than for older adults due to differences in immunogenicity by age group [31]. Cromer and colleagues estimated variant-specific efficacy against symptomatic and severe infection and also modeled boosting impact after primary series vaccination [15]. This study did not estimate efficacy by different vaccines and vaccine protection against SARS-CoV-2 infection between different time periods after primary series vaccination. Furthermore, the influence of age was not considered in the study, thus, age-specific efficacy estimates from different vaccines were not made. In contrast, our study provides a timely prediction of vaccine efficacy against both the prototype strain and the Delta variant across different vaccines, clinical endpoints, and time since primary series vaccination, as well as predicting impact of homologous third doses. Our study confirmed that timely vaccination of the prototype-based COVID-19 vaccines can effectively protect against severe outcomes caused by SARS-CoV-2 variant infections. Two other studies determined protective thresholds against different severities for mRNA-1273 and ChAdOx1nCoV-19. Combined with the results of our study, these data provided valuable evidence about duration of effective protection after primary and homologous boosting vaccination with these two vaccines [32, 33].

Our study has several limitations. First, our predictions depend on level of neutralizing antibodies, without taking into account other immunologic mechanisms of humoral and cellular immunity. However, previous studies showed that neutralizing antibodies are highly predictive of immune protection. Second, to enhance comparability between different studies with different neutralization assays, we normalized the neutralization titers in each study by the mean titers in convalescent sera reported in phase I/II trials against prototype strains that used the same type of neutralization assay. However, other sources of heterogeneity, such as lab-to-lab variation and experimental procedures could not be accounted for. We could not control for potential biases or uncertainties caused by heterogeneity of convalescent sera. Further studies are recommended to use World Health Organization (WHO) standardized human convalescent sera as a validated positive control and to calibrate antibody results by using WHO International Units to make immunogenicity of different COVID-19 vaccine candidates more comparable. Lastly, due to a paucity of studies that evaluated long-term antibody dynamics after homologous boosting dose, we could not predict corresponding vaccine efficacy or evaluate time until immunity is lost. Further studies will be needed to address these questions.

## Conclusions

In conclusion, our study predicted time-varying vaccine protection from prototype SARS-CoV-2 and the Delta variant and the increase of protection that results from homologous third dose administration. We estimated protection by four COVID-19 vaccines from three clinical endpoints in two age groups. Our findings suggest that, regardless of age group, timely boosting with SARS-CoV-2-prototype-based vaccines can provide protection against the Delta variant, with better performance from mRNA vaccines than from protein and inactivated vaccines. Irrespective of vaccine technology, however, third doses will effectively prevent symptomatic and severe COVID-19 caused by the Delta variant. Long-term monitoring and surveillance of antibody dynamics and vaccine protection, as well as further validation of neutralizing antibody or other markers that can serve as correlates of protection, are urgently needed to inform COVID-19 pandemic responses.

## Supplementary Information

**Additional file 1: Table S1.** Baseline demographic characteristics for the study population. **Table S2.** Comparison of SARS-CoV-2 neutralizing antibody levels between adults, 18-59 years of age and elderly adults, 60 years and older. **Table S3.** Neutralizing antibody dynamics for CoronaVac by time period. **Table S4.** Search terms for the three peer-reviewed databases and two preprint servers. **Table S5.** Inclusion and exclusion criteria for systematic review. **Table S6.** Summary of neutralizing antibody levels over time by different vaccines. **Table S7.** Comparison of neutralization assays used in homologous prime-boost studies and in phase I/II clinical trials for convalescent sera. **Table S8.** Fold-changes of neutralization antibody levels against the Delta variant compared to the prototype strain. **Figure S1.** Times of blood sample collection. **Figure S2.** CoronaVac vaccine-induced SARS-CoV-2 antibody titers and seroprevalence by age group and dose number. **Figure S3.** Fitted profiles for CoronaVac vaccine-induced SARS-CoV-2 antibody titers and seroprevalence by age after the first and second doses. **Figure S4.** Dynamic patterns of CoronaVac vaccine-induced SARS-CoV-2 antibody titers and corresponding seroprevalences after the first and second doses on days 0 and 28. **Figure S5.** Loss of protective immunity acquired from two doses of CoronaVac vaccine. **Figure S6.** Predicted CoronaVac vaccine-induced neutralizing antibody titers against the Delta SARS-CoV-2 variant in vaccine recipients, assuming 2.4-fold reduction of antibody levels against the Delta SARS-CoV-2 variant compared to the prototype SARS-CoV-2 strain. **Figure S7.** Flowchart of inclusion of retrieved studies. **Figure S8.** Predicting efficacy over time across different vaccines and clinical endpoints for other VOCs. **Figure S9.** Sensitivity analysis of predicted efficacy changing fold-change parameter for mRNA-1273. **Figure S10.** Sensitivity analysis of predicted efficacy by using upper limit of model parameter slope (k). **Figure S11.** Sensitivity analysis of predicted efficacy by using lower limit of model parameter slope (k).

## Data Availability

All datasets generated and analyzed are available in the article and Additional file 1.

## Abbreviations

COVID-19: Coronavirus disease 2019
SARS-CoV-2: Severe acute respiratory syndrome coronavirus 2
GMTs: Geometric mean titers
GAM: Generalized additive model
BIC: Bayesian information criteria
AIC: Akaike information criteria
MCMC: Markov Chain Monte Carlo
VOC: Variant of concern
WHO: World Health Organization
