# Supplementary material for: Prediction of long-term kinetics of vaccine-elicited neutralizing antibody and time-varying vaccine-specific efficacy against the SARS-CoV-2 Delta variant by clinical endpoint
Source: BMC Med. 2022 Jan 28;20:36. doi: 10.1186/s12916-022-02249-9 (PMC8794738; doi:10.1186/s12916-022-02249-9)
Supplement: Supplementary file 1 — Additional file 1: Table S1. Baseline demographic characteristics for the study population. Table S2. Comparison of SARS-CoV-2 neutralizing antibody levels between adults, 18-59 years of age and elderly adults, 60 years and older. Table S3. Neutralizing antibody dynamics for CoronaVac by time period. Table S4. Search terms for the three peer-reviewed databases and two preprint servers. Table S5. Inclusion and exclusion criteria for systematic review. Table S6. Summary of neutralizing antibody levels over time by different vaccines. Table S7. Comparison of neutralization assays used in homologous prime-boost studies and in phase I/II clinical trials for convalescent sera. Table S8. Fold-changes of neutralization antibody levels against the Delta variant compared to the prototype strain. Figure S1. Times of blood sample collection. Figure S2. CoronaVac vaccine-induced SARS-CoV-2 antibody titers and seroprevalence by age group and dose number. Figure S3. Fitted profiles for CoronaVac vaccine-induced SARS-CoV-2 antibody titers and seroprevalence by age after the first and second doses. Figure S4. Dynamic patterns of CoronaVac vaccine-induced SARS-CoV-2 antibody titers and corresponding seroprevalences after the first and second doses on days 0 and 28. Figure S5. Loss of protective immunity acquired from two doses of CoronaVac vaccine. Figure S6. Predicted CoronaVac vaccine-induced neutralizing antibody titers against the Delta SARS-CoV-2 variant in vaccine recipients, assuming 2.4-fold reduction of antibody levels against the Delta SARS-CoV-2 variant compared to the prototype SARS-CoV-2 strain. Figure S7. Flowchart of inclusion of retrieved studies. Figure S8. Predicting efficacy over time across different vaccines and clinical endpoints for other VOCs. Figure S9. Sensitivity analysis of predicted efficacy changing fold-change parameter for mRNA-1273. Figure S10. Sensitivity analysis of predicted efficacy by using upper limit of model parameter slope (k). Figure S11. Se [file 12916_2022_2249_MOESM1_ESM.docx]

**Additional file**

[Neutralizing antibody dynamics for CoronaVac from individual titer data	1](#_Toc82766124)

[1. Baseline characteristics of participants received CoronaVac vaccine	1](#_Toc82766125)

[Table S1. Baseline demographic characteristics for the study population.	1](#_Toc82766126)

[Figure S1. Time distribution of blood sample collection**.**	2](#_Toc82766127)

[2. Generalized addictive model (GAM) for neutralization data fitting	2](#_Toc82766128)

[3. Multivariate Imputation procedure reducing curve-fitting uncertainty of the antibody titers kinetics	2](#_Toc82766129)

[4. Observed CoronaVac vaccine-induced SARS-CoV-2 antibody titers against prototype SARS-CoV-2 strain	3](#_Toc82766130)

[Figure S2. CoronaVac vaccine-induced SARS-CoV-2 antibody titers and seroprevalence by age group and dose of vaccination.	4](#_Toc82766131)

[Table S2. Comparison of SARS-CoV-2 neutralizing antibody between adults and elderly.	4](#_Toc82766132)

[Table S3. Neutralizing antibody dynamics for CoronaVac by time periods	6](#_Toc82766133)

[5. Kinetics of CoronaVac vaccine-induced SARS-CoV-2 antibody titers against prototype SARS-CoV-2 strain	7](#_Toc82766134)

[Figure S3. Fitted profiles for CoronaVac vaccine-induced SARS-CoV-2 antibody titers and seroprevalence by age after the first or second dose of vaccine.	7](#_Toc82766135)

[Figure S4. Dynamic pattern of CoronaVac vaccine-induced SARS-CoV-2 antibody titers and the corresponding seroprevalence after the first and second doses of vaccine on days 0 and 28.	8](#_Toc82766136)

[6. Half-life of CoronaVac vaccine-induced SARS-CoV-2 antibody	8](#_Toc82766137)

[Figure S5. Loss of protective immunity acquired from two doses of CoronaVac vaccine.	8](#_Toc82766138)

[7. Kinetics of CoronaVac vaccine-induced SARS-CoV-2 antibody titers against Delta SARS-CoV-2 variant	9](#_Toc82766139)

[Figure S6. Predicted CoronaVac vaccine-induced neutralizing antibody titer against Delta SARS-CoV-2 variant in vaccine recipients, assuming 2.4-fold reductions of antibody levels in Delta SARS-CoV-2 variant compared to prototype SARS-CoV-2 strain.	9](#_Toc82766140)

[Neutralizing antibody dynamics across different vaccines from a comprehensive literature search	10](#_Toc82766141)

[1. Literature search for neutralizing antibody dynamics induced by different vaccines	10](#_Toc82766142)

[Table S4. Search terms for three peer-reviewed databases and two preprint servers.	10](#_Toc82766143)

[2. Summary of retrieved data of neutralizing antibody dynamics for different vaccines.	12](#_Toc82766144)

[Table S5. Summary of level of neutralizing antibody over time by different vaccines	12](#_Toc82766145)

[*In vitro* cross neutralization titer against both prototype and Delta variant	14](#_Toc82766146)

[Table S6. Fold change of neutralization antibody level of the Delta variant compared to prototype strains	15](#_Toc82766147)

[Predicting vaccine efficacy over time across different vaccines and clinical endpoints	16](#_Toc82766148)

[1. Sensitivity analysis of changing fold-change parameter for mRNA-1273	16](#_Toc82766149)

[Figure S7. Sensitivity analysis of changing fold-change parameter for mRNA-1273	17](#_Toc82766150)

[References	18](#_Toc82766151)

# Neutralizing antibody dynamics for CoronaVac from individual titer data

## 1. Baseline characteristics of participants received CoronaVac vaccine

Immunogenicity data in phase 1/2 clinical trial of CoronaVac from a total of 244 healthy adults who were intramuscularly administered CoronaVac vaccine of 3 μg at days 0 and 28 were used to evaluate the long-term kinetics of CoronaVac vaccine-induced SARS-CoV-2 antibody titers. Details of baseline demographic characteristics and time distribution of blood sample collection for 244 study participants are shown in **Table S1** and **Fig. S1**. The median age of these participants is 60 (range: 18-59) years, and 50.4% of them (n=123) are male.

### Table S1. Baseline demographic characteristics for the study population.

| Characteristics | Overall  (n=244) | Adults aged 18-59 years  (n=120) | Elderly aged 60 years or older  (n=124) |
| --- | --- | --- | --- |
| Sex |  |  |  |
| Female | 121 (49.6) | 57 (23.4) | 64 (26.2) |
| Male | 123 (50.4) | 63 (25.8) | 60 (24.6) |
| Age, years |  |  |  |
| Median (Range) | 60 (18-89) | 44 (18-59) | 65.5 (60-89) |
| 19-29 | 17 (7.0) | 17 (14.2) | 0 (0) |
| 30-39 | 24 (9.8) | 24 (20.0) | 0 (0) |
| 40-49 | 52 (21.3) | 52 (43.3) | 0 (0) |
| 50-59 | 27 (11.1) | 27 (22.5) | 0 (0) |
| ≥60 | 124 (50.8) | 0 (0) | 124 (100) |

### Figure S1. Times of blood sample collection**.**

(A) Time distribution of blood sample collection in adults; (B) Time distribution of blood sample collection in elderly. Note that red arrows in each panel refer to the date of second-dose vaccination.

## 2. Generalized addictive model (GAM) for neutralization data fitting

The specification for the GAM smoothing in a Gaussian model is defined as follows:

$$g\left[ E\left( y_{i} \right) \right]=\alpha+\beta\cdot s(x_{i})$$

where $E(y_{i})$ is mean neutralizing antibody titer against SARS-CoV-2, g is the log link function,$\alpha$ is the intersect, $s(x_{i})$ is the value at a given time $x_{i}$ of a smoothing spline basis, and $\beta$ is the vector of spline coefficient.

We also fit seropositivity rate across time by using generalized addictive models. The specifications of GAM models are defined as follows:

$$g(u_{i})=\alpha+\beta s(x_{i})$$

where g is a log link function, $u_{i}=E(Y)$ is the mean of the response variable $y_{i}$, $\alpha$ is the intersect, $s(x_{i})$ is spline for a given time $x_{i}$ and $\beta$ is the vector of spline coefficients.

## 3. Multivariate Imputation procedure reducing curve-fitting uncertainty of the antibody titers kinetics

Considering the large uncertainties of fit curves of the antibody titers kinetics which is attributed to a lack of observed immunogenicity data during 120-209 days after first-dose CoronaVac vaccination, we obtained imputed data from a state-of-the-art Multivariate Imputation by the pan algorithm which allows for Markov Chain Monte Carlo (MCMC) techniques to draw replacements for multilevel missing values in the database, using antibody titers data that collected from participants received other dosing schedules in phase 1/2 clinical trial of CoronaVac. The number of multiple imputations was set as 5 with each imputation running 5 realizations. For each of the 5 realizations of imputed databases, we independently performed regression analysis of antibody titer concentration, using age and sex of participants, interval between the first- and second-doses of CoronaVac vaccine, the level of the spike antigen contained in the vaccine, the number of doses received, as well as the random effects attributed to a participant during each follow-up visit, which are key determinants for the distribution of antibody titer concentration.

## 4. Observed CoronaVac vaccine-induced SARS-CoV-2 antibody titers against prototype SARS-CoV-2 strain

Consistent with our previously qualitative analyses of CoronaVac vaccine-induced SARS-CoV-2 antibody titers, we also estimated the geometric mean titer (GMT) and the seroprevalence of neutralizing antibodies against SARS-CoV-2 among 244 study participants. The 95% confidence interval (CIs) for the estimates of the seroprevalence of SARS-CoV-2 neutralizing antibody were calculated on the assumption of binomial distribution with normal approximation. Results suggested that GMTs is higher than the protective threshold of 33 at days 56-89 after the first dose of CoronaVac vaccine, with more than 50% of participants that are being protective, regardless of the age of participants (**Fig. S2** and **Table S2**). Moreover, we observed that GMTs and the seroprevalence increased significantly when one booster dose was administered to participants.

### Figure S2. CoronaVac vaccine-induced SARS-CoV-2 antibody titers and seroprevalence by age group and dose of vaccination.

(A-B) Observed geometric mean titers and seroprevalence in all participants received two primary doses of CoronaVac vaccine; (C-D) Observed geometric mean titers and seroprevalence in adults and elderly received two primary doses of CoronaVac vaccine; (E-F) Observed geometric mean titers and seroprevalence in all participants who received two primary doses plus one booster dose of CoronaVac vaccine.

### Table S2. Comparison of SARS-CoV-2 neutralizing antibody between adults, 18-59 years of age and elderly adults 60 years and older.

| Time since first dose of vaccination | Total no. of serums | Total no. of participants | No. of seropositivity | GMTs  (95%CI) | P-value^a^ | Seroprevalence  (%, 95%CI) | P-value^b^ |
| --- | --- | --- | --- | --- | --- | --- | --- |
| Overall | | | | | | | |
| <14 days | 244 | 244 | 0 | 2.0 (2.0, 2.0) | - | 0 (0, 1.5) | - |
| 28-55 days | 24 | 24 | 1 | 6.9 (4.7, 10.0) | <0.001 | 4.2 (0.1, 21.1) | 0.077 |
| 56-89 days | 241 | 241 | 138 | 43.7 (38.9, 49.1) | <0.001 | 57.3 (50.8, 63.6) | <0.001 |
| 7 months | 173 | 173 | 7 | 4.4 (3.8, 5.1) | <0.001 | 4.0 (1.6, 8.2) | <0.001 |
| Adults aged 18-59 years | | | | | | | |
| <14 days | 120 | 120 | 0 | 2.0 (2.0, 2.0) | - | 0 (0, 3) | - |
| 56-89 days | 117 | 117 | 66 | 44.1 (37.2, 52.2) | <0.001 | 56.4 (46.9, 65.6) | <0.001 |
| 7 months^c^ | 54 | 54 | 3 | 6.7 (5.2, 8.6) | <0.001 | 5.6 (1.2, 15.4) | <0.001 |
| Elderly aged ≥60 years | | | | | | | |
| <14 days | 124 | 124 | 0 | 2.0 (2.0, 2.1) | - | 0 (0, 2.9) | - |
| 28-55 days | 24 | 24 | 1 | 6.9 (4.7, 10) | <0.001 | 4.2 (0.1, 21.1) | 0.162 |
| 56-89 days | 124 | 124 | 72 | 43.4 (36.9, 51) | <0.001 | 58.1 (48.9, 66.9) | <0.001 |
| 7 months^c^ | 119 | 119 | 4 | 3.7 (3.1, 4.3) | <0.001 | 3.4 (0.9, 8.4) | <0.001 |

^a^ Difference in GMTs between two adjacent time intervals;

^b^ Difference in the seroprevalence of SARS-CoV-2 antibody titers between two adjacent time intervals;

^c^ GMTs is significantly higher in adults aged 18-59 years than those in elderly aged ≥60 years

### Table S3. Neutralizing antibody dynamics for CoronaVac by time period

| **Reference** | **Vaccine** | **Age** | **No. participants** | **Administration regimen** | **Neutralization assay** | **GMT (95%CI) *** | **Neutralizing titer of convalescent (Phase I/II trials)** |
| --- | --- | --- | --- | --- | --- | --- | --- |
| SinoVac | CoronaVac | ≥18 yrs |  | Dose 1 & 2: 3μg with 28 days apart;  Dose 3: 8 months after the second dose | CPE-based microneutralization assay | 14-30 d after the second dose: 37.3 (33.3-41.8)  6-8 months after the second dose: 5.2 (4.6-5.9)  14-30 d after dose 3: 147.0 (117.5-183.9) | 163.7 |
|  |  | 18-55 yrs |  | Dose 1 & 2: 3μg with 28 days apart;  Dose 3: 8 months after the second dose | CPE-based microneutralization assay | 14-30 d after the second dose: 43.7 (36.8-51.9)  6-8 months after the second dose: 8.2 (6.7-10.1)  14-30 d after dose 3: 137.9 (108.6-175.1) | 163.7 |
|  |  | 65-85 yrs |  | Dose 1 & 2: 3μg with 28 days apart;  Dose 3: 8 months after the second dose | CPE-based microneutralization assay | 14-30 d after the second dose: 45.7 (37.7-55.3) 6-8 months after the second dose: 3.9 (3.1-4.9) 14-30 d after dose 3: 158.4 (88.9-282.3) | 163.7 |

## 5. Kinetics of CoronaVac vaccine-induced SARS-CoV-2 antibody titers against prototype SARS-CoV-2 strain

We evaluate the kinetics of CoronaVac vaccine-induced SARS-CoV-2 antibody titers among participants who were intramuscularly administered CoronaVac vaccine of 3 μg at days 0 and 28. We found that, from day 20 to day 25, detectable antibody titers against prototype SARS-CoV-2 strain were observed among CoronaVac vaccine recipients, and they had detectable antibody titers against SARS-CoV-2 up to 5.3 months after the second dose of CoronaVac vaccine (**Fig. S3**).

### Figure S3. Fitted profiles for CoronaVac vaccine-induced SARS-CoV-2 antibody titers and seroprevalence by age after the first or second dose of vaccine.

(A) Fitted SARS-CoV-2 antibody titer dynamics in all participants; (B) Fitted SARS-CoV-2 antibody titer dynamics by age group; (C) Fitted seroprevalence in all participants (with a protective threshold of 33 for seropositivity); (D) Fitted seroprevalence by age group (with a protective threshold of 33 for seropositivity).

We also assessed the kinetics of CoronaVac vaccine-induced SARS-CoV-2 antibody titers among participants who timely received two primary doses of CoronaVac vaccine at days 0 and 28, and found that the time to reach and to decrease below the protective threshold did not change (**Fig. S4**).

### Figure S4. Dynamic pattern of CoronaVac vaccine-induced SARS-CoV-2 antibody titers and the corresponding seroprevalence after the first and second doses of vaccine on days 0 and 28.

(A) Fitted SARS-CoV-2 antibody titer dynamics; (B)Fitted seroprevalence (with a protective threshold of 33 for seropositivity).

## 6. Half-life of CoronaVac vaccine-induced SARS-CoV-2 antibody

### Figure S5. Loss of protective immunity acquired from two doses of CoronaVac vaccine.

(A) Kaplan-Meier plot of probability of seropositivity (titer of 33 or higher) in all participants; (B) Cumulative probability of seronegativity in all participants; (C) Kaplan-Meier plot of probability of seropositivity by age group; (D) Cumulative probability of seronegativity by age group.

## 7. Kinetics of CoronaVac vaccine-induced SARS-CoV-2 antibody titers against Delta SARS-CoV-2 variant

### Figure S6. Predicted CoronaVac vaccine-induced neutralizing antibody titer against Delta SARS-CoV-2 variant in vaccine recipients, assuming 2.4-fold (95% CI: 1.1-5.2 fold) reductions of antibody levels in Delta SARS-CoV-2 variant compared to prototype SARS-CoV-2 strain.

(A) Overall; (B) Adults aged 18-59 years of age; (C) Elderly aged 60 years or over.

# Neutralizing antibody dynamics across different vaccines from a comprehensive literature search

## 1. Literature search for neutralizing antibody dynamics induced by different vaccines

We conducted a systematic search from five databases, including three peer-reviewed databases (PubMed, Embase and Web of Science) and two preprint servers (medRxiv, bioRxiv), for studies that reported dynamics of neutralizing antibodies and impact of a booster dose among vaccine recipients with predefined search terms **(Table S4)**. Besides, we collected official reports from vaccine companies to supplement our retrieved data.

### Table S4. Search terms for three peer-reviewed databases and two preprint servers.

| **Database** | **Step** | **Search strategy** | **Number of articles*** |
| --- | --- | --- | --- |
| PubMed | #1 | “COVID-19 Vaccines”[Mesh] | 6,718 |
|  | #2 | COVID-19[tiab] AND vaccin*[tiab] | 17,426 |
|  | #3 | #1 OR #2 | 19,168 |
|  | #4 | ((“BNT162b2” OR “Pfizer-BioNTech” OR “Pfizer/BioNTech” OR “Comirnaty”) OR (“mRNA-1273” OR “Moderna COVID-19 vaccine”) OR (“ChAdOx1 nCoV-19” OR “AZD1222” OR “ChAdOx1-S” OR “Oxford-AstraZeneca” OR “Covishield”) OR (“Gam-COVID-Vac” OR “Sputnik V”) OR (“Ad5 nCoV” OR “Cansino”) OR (“BBIBP-CorV” OR “WBIP” OR “Sinopharm”) OR (“CoronaVac” OR “SinoVac”) OR (“BBV152” OR “Covaxin”) OR (“Ad26.COV2.S” OR “Janssen COVID-19 Vaccine”) OR “ZF2001”)) | 2,745 |
|  | #5 | #3 OR #4 | 19,827 |
|  | #6 | “Antibodies, Neutralizing”[Mesh] | 14,040 |
|  | #7 | “neutralizing antibodies”[tiab] OR “neutralizing antibody”[tiab] OR neutralizing[tiab] OR neutralization[tiab] | 82,871 |
|  | #8 | (“Antibodies, Neutralizing”[Mesh] OR “neutralizing antibodies”[tiab] OR “neutralizing antibody”[tiab] OR neutralizing[tiab] OR neutralization[tiab]) | 85,717 |
|  | #9 | (dynamic[tiab] OR dynamics[tiab] OR kinetic[tiab] OR kinetics[tiab] OR durable[tiab] OR duration[tiab] OR durability[tiab]OR persistence[tiab] OR persisting[tiab] OR longitudinal[tiab] OR waning[tiab] OR long-term[tiab] OR short-term[tiab]) | 3,094,355 |
|  | #10 | #5 AND #8 AND #9 | 373 |
| Embase | #1 | Title or Abstract=(COVID-19 AND vaccin*) | 15,405 |
|  | #2 | Title or Abstract=(("BNT162b2" OR "Pfizer-BioNTech" OR “Pfizer/BioNTech" OR “Comirnaty") OR ("mRNA-1273" OR "Moderna COVID-19 vaccine") OR ("ChAdOx1 nCoV-19" OR "AZD1222" OR "ChAdOx1-S" OR "Oxford-AstraZeneca" OR "Covishield") OR ("Gam-COVID-Vac" OR "Sputnik V") OR ("Ad5 nCoV" OR "Cansino") OR ("BBIBP-CorV" OR "WBIP" OR "Sinopharm") OR ("CoronaVac" OR "SinoVac") OR ("BBV152" OR "Covaxin") OR ("Ad26.COV2.S" OR "Janssen COVID-19 Vaccine") OR "ZF2001") | 2,160 |
|  | #3 | #1 OR #2 | 16,036 |
|  | #4 | Title or Abstract=("neutralizing antibodies" OR "neutralizing antibody" OR neutralizing OR neutralization) | 98,725 |
|  | #5 | Title or Abstract=((dynamic OR dynamics OR kinetic OR kinetics OR durable OR duration OR durability OR persistence OR persisting OR longitudinal OR waning OR long-term OR short-term)) | 3,963,517 |
|  | #6 | #3 AND #4 AND #5 | 262 |
| Web of Science | #1 | TS=(COVID-19 AND vaccin*) | 24,359 |
|  | #2 | TS=((((“BNT162b2” OR “Pfizer-BioNTech” OR “Pfizer/BioNTech” OR “Comirnaty”) OR (“mRNA-1273” OR “Moderna COVID-19 vaccine”) OR (“ChAdOx1 nCoV-19” OR “AZD1222” OR “ChAdOx1-S” OR “Oxford-AstraZeneca” OR “Covishield”) OR (“Gam-COVID-Vac” OR “Sputnik V”) OR (“Ad5 nCoV” OR “Cansino”) OR (“BBIBP-CorV” OR “WBIP” OR “Sinopharm”) OR (“CoronaVac” OR “SinoVac”) OR (“BBV152” OR “Covaxin”) OR (“Ad26.COV2.S” OR “Janssen COVID-19 Vaccine”) OR “ZF2001”))) | 2,873 |
|  | #3 | #1 OR #2 | 24,749 |
|  | #4 | TS=("neutralizing antibodies" OR "neutralizing antibody" OR neutralizing OR neutralization) | 329,559 |
|  | #5 | TS=((dynamic OR dynamics OR kinetic OR kinetics OR durable OR duration OR durability OR persistence OR persisting OR longitudinal OR waning OR long-term OR short-term)) | 12,949,001 |
|  | #6 | #3 AND #4 AND #5 | 518 |
| bioRxiv & medRxiv | #1 | COVID-19 AND vaccin* | 7,713 |
|  | #2 | neutralizing antibody | 2,617 |
|  | #3 | dynamics OR kinetics OR longitudinal | 115,688 |
|  | #4 | #1 AND #2 AND #3 | 1,088 |

* The search was done on Nov 26, 2021.

### Table S5. Inclusion and exclusion criteria for systematic review.

**Inclusion criteria**

1. Studies reported vaccine-elicited neutralizing antibodies tiers or GMT of prototype strains overtime after both primary and boost vaccination among COVID-19 naïve population were included.

2. The type of neutralization assay must be consistent with the assay used in phase I/II clinical trials of the vaccine.

3. The neutralization data for COVID-19 convalescents must be available in phase I/II clinical trials of the vaccine.

**Exclusion criteria**

1. Non-neutralization data were excluded (e.g. IgG, IgA).

2. Studies only reported neutralizing antibody response after primary vaccination or booster dose were excluded.

3. Studies only reported vaccine-elicited neutralizing antibody response without longitudinal follow-up were excluded.

4. Neutralization titer from animal assay were excluded.

5. Studies report vaccine-elicited neutralizing antibody response among specific populations (e.g. COVID-19 patients, HIV patients, pregnancy) were excluded.

6. Abstracts of congress meetings or conference proceedings, study protocols, media news, commentaries, reviews or case reports were excluded.

### Figure S7. Flowchart of inclusion of retrieved studies.

* Eight other records were excluded, including 1 review, 2 animal experiments, 2 heterologous prime-boost studies, 2 cellular-immunity studies, and 1 neutralization-assay methodology study.

## 2. Summary of retrieved data of neutralizing antibody dynamics for different vaccines.

### Table S6. Summary of neutralizing antibody levels over time by different vaccines

| **Reference** | **Vaccine** | **Age** | **No. participants** | **Administration regimen** | **Neutralization assay** | **GMT (95%CI) *** | **Neutralizing titer of convalescent (Phase I/II trials)** | **Data sources** |
| --- | --- | --- | --- | --- | --- | --- | --- | --- |
| Pfizer | BNT162b2 | 18-55 yrs | 11 | Dose 1 & 2: 30mcg BNT162b2 with 21 days apart;  Dose 3: >6 months. after 2nd dose of BNT162b2 | Plaque reduction neutralizing test, PRNT50 | Day 1 pre-vaccination: 10  Day 7 post dose 2: 497 (247.88-978.65)  Month 1 post dose 2: 387 (245.02-591.59)  Month 8 post dose 2: 83 (34.83-193.85)  Day 7 post dose 3: 1754 (1260.21-2504.06)  Month 1 post dose 3: 2119 (1257.3-3393.18) | 94 | Falsey et al., SARS-CoV-2 Neutralization with BNT162b2 Vaccine Dose 3, *NEJM*;  Walsh et al., Safety and Immunogenicity of Two RNA-Based Covid-19 Vaccine Candidates, *NEJM* |
|  | BNT162b2 | 65-85 yrs | 12 | Dose 1 & 2: 30mcg BNT162b2 with 21 days apart;  Dose 3: >6 months. after 2nd dose of BNT162b2 | Plaque reduction neutralizing test, PRNT50 | Day 1 pre-vaccination: 10  Day 7 post dose 2: 538 (288.82-982.99)  Month 1 post dose 2: 261 (153.32-425.48)  Month 8 post dose 2: 41 (24.14-70.82)  Day 7 post dose 3: 1318 (889.68-1751.43)  Month 1 post dose 3: 2032 (1228.18-3253.67) | 94 |  |
|  | BNT162b2^#^ | 18-85 yrs | 23 | Dose 1 & 2: 30mcg BNT162b2 with 21 days apart;  Dose 3: >6 months. after 2nd dose of BNT162b2 | Plaque reduction neutralizing test, PRNT50 | Day 1 pre-vaccination: 10  Day 7 post dose 2: 502 (329-765)  Month 1 post dose 2: 295 (209-417)  Month 8 post dose 2: 55 (34-90)  Day 7 post dose 3: 1464 (1134-1890)  Month 1 post dose 3: 1990 (1416-2794) | 94 |  |
| Moderna | mRNA-1273 | ≥18 yrs | 20 | Dose 1 & 2: 100mcg mRNA-1273 with 28 days apart;  Dose 3: 50mcg mRNA-1273 after 2nd dose | Pseudovirus neutralization assay, ID50 | Day 1 pre dose1: 107  Day 29 post dose 1: 1786  Month 1 post dose 2: 1210 (839.88-1739.68)  Month 6-8 post dose 2: 198 (125.66-314.51)  2 weeks after booster: 4588 (3314.64-6572.12) | 109.2 | Moderna 2Q 2021 Earnings Call [https://investors.modernatx.com/events/event-details/moderna-2q-2021-earnings-call]  Choi et al., Safety and immunogenicity of SARS-CoV-2 variant mRNA vaccine boosters in healthy adults: an interim analysis. *Nature medicine*.  Jackson et al., An mRNA Vaccine against SARS-CoV-2 - Preliminary Report, *NEJM* |
| NOVAVAX | NVX-CoV2373 | 18-84 yrs | 258 | Dose 1 & 2: 5μg protein + Matrix-M with 21 days apart;  Dose 3: 189 days after 1st dose of NVX-CoV2373 | Live virus microneutralization assay IC50 | Day 0 pre-vaccination: 10  Day 0 before dose 2: 33 (10.74-93.32)  Day 14 post dose 2: 1581 (1029.68-2379.27)  Day 0 before dose 3: 65 (40.39-99.47)  Day 28 post dose 3: 6039 (4432.53-7998.39) | 453 (259-700)^£^ | Second Quarter 2021 Financial Results and Operational Highlights [https://filecache.investorroom.com/mr5ir_novavax/115/Q2-2021-Earnings-Slides.pdf]  Formica et al., Different dose regimens of a SARS-CoV-2 recombinant spike protein vaccine (NVX-CoV2373) in younger and older adults: A phase 2 randomized placebo-controlled trial. PLoS Med |
| Livzon | V01 | 18-59 yrs | 23 | Dose 1 & 2: 10 μg with 21 apart  Dose: 4-5 months after the second dose | Live SARS-CoV-2 neutralization assay | Day 14 post dose 2: 110 (85-141)  Day 0 before dose 3: 17 (12-23)  Day 14 post dose 3: 1017 (732-1413)  Day 28 post dose 3: 765 (608-962) | 53.6 | Li Y et al., Immunogenicity and safety of the homogenous booster shot of a recombinant fusion protein vaccine (V-01) against COVID-19 in healthy adult participants primed with a two-dose regimen.  Shu Y-J et al., Immunogenicity and safety of a recombinant fusion protein vaccine (V-01) against coronavirus disease 2019 in healthy adults: a randomized, double-blind, placebo-controlled, phase II trial. *Chinese medical journal* 2021 |
|  | V01 | ≥60 yrs | 20 | Dose 1 & 2: 10 μg with 21 apart  Dose: 4-5 months after the second dose | Live SARS-CoV-2 neutralization assay | Day 14 post dose 2: 113 (66-195)  Day 0 before dose 3: 14 (9-20)  Day 14 post dose 3: 729 (397-1339)  Day 28 post dose 3: 697 (416-1165) | 53.6 |  |
|  | V01 | ≥18 yrs | 43 | Dose 1 & 2: 10 μg with 21 apart  Dose: 4-5 months after the second dose | Live SARS-CoV-2 neutralization assay | Day 0 pre-vaccination: 5 (-)  Day 0 before dose 2: 15 (10-22)  Day 14 post dose 2: 111 (83-146)  Day 28 post dose 2: 116 (83-150)  Day 0 before dose 3: 15 (11-19)  Day 14 post dose 3: 871 (604-1153)  Day 28 post dose 3: 733 (539-895) | 53.6 |  |

* For studies that did not report 95% confidence intervals, we digitized individual titers from figures using a digital extraction tool and calculated the intervals.

\# Combined data set for different age group.

£ Neutralization titer of convalescent patients aged 18-59 years of age was used.

### Table S7. Comparison of neutralization assay used in homologous prime-boost studies and in phase I/II clinical trials for convalescent sera

| **Vaccine** | **Neutralization assay in** **homologous prime-boost studies** | | | | | **Neutralization assay for convalescents in Phase I/II clinical trials** | | | | | | | | |
| --- | --- | --- | --- | --- | --- | --- | --- | --- | --- | --- | --- | --- | --- | --- |
|  | Serologic assay | Virus | Cell | Serum | Readout | Serologic assay | Samples | Virus | Cell | Serum | Readout | LOD | GMT | Note |
| BNT162b2 | Plaque-reduction neutralization test | 100 PFU of Recombinant USA-WA1/2020 | Vero E6 cells | 2-fold serially diluted (1:40 to 1:1280); | A minimal serum dilution that suppressed >50% of viral plaques is defined as PRNT50 | Fluorescence-based neutralization assay | 38 convalescent serum samples were collected at least 14 days after PCR-confirmed diagnosis and at  a time when the donors were asymptomatic. | mNG SARS-CoV-2 (the mNG gene was engineered at the ORF7 of the viral genome) | Vero CCL81 cell | Serially diluted and incubated with the reporter virus. After incubation at 37°C for 1 h | Dilution fold that neutralized  50% of fluorescent cells (NT50). | 1:20 (2 were below the lower limits) | 94 | The fluorescent neutralization assay produced comparable results as the conventional plaque reduction  neutralization assay. |
| mRNA-1273 | VSV-based pseudovirus neutralization assay | SARS-CoV-2 full-length S pseudotyped recombinant  VSV-ΔG-firefly luciferase virus | A549-hACE2-TMPRSS2 cells | Serially diluted serum samples were mixed with pseudovirus and incubated  at 37 °C for 45 min. | Neutralization is measured as the serum dilution at which the RLUs are reduced by 50% (ID50) | VSV-based pseudovirus neutralization assay | 38 convalescent serum samples | A pseudotyped virus particles bearing  codon-optimized SARS-CoV-2 full-length S plasmid | ACE-2-overexpressing 293T cells | Serially diluted with the initial dilution of 1:20. | Neutralization is measured as the serum dilution at which the RLUs are reduced by 50% (ID50) | 1:20 (8 were below the lower limits and expressed as 1:10) | 109.2 | - |
| MVX-CoV2373 | Wild-type microneutralization assay | - | - | - | IC50 | Wild-type microneutralization assay | 60 convalescent sera samples (3 from pediatric cases, 13 were adults 18-49 years, 13 were adults 50-64 years, and 31 were adults 65-79 years; 18% were asymptomatic/mild cases, 30% were moderated cases,32% were severe cases) | SARS-CoV-2 hCoV-19/Australia/VIC01/2020 | Vero E6 cells | All serum samples were heat inactivated at 56°C for 30 minutes. | A microneutralization assay with an inhibitory  concentration of 50% was used to measure neutralizing antibodies (IC50) | 1:20 (none were below the lower limit) | 453 | - |
| CoronaVac | CPE-based live SARS-CoV-2 microneutralization assay | Wild-type SARS-CoV-2 (100CCID50/0.05ml) | Vero cell | Four-fold serial diluted (inactivated at 56℃ in a water bath for 30 minutes) | The reciprocal of the highest serum dilution without cytopathic effects the end titer. | CPE-based live SARS-CoV-2 microneutralization assay | 117 convalescent serum samples collected from 70 patients | Wild-type SARS-CoV-2 (100CCID50/0.05ml) | Vero cell | Serially diluted with the initial dilution of 1:4. | The reciprocal of the highest serum dilution without cytopathic effects the end titer. | 1:4 | 163.7 | The neutralization assay used in Phase I/II trials was the same as that used in homologous prime-boost studies study |
| V01 | Live SARS-CoV-2 neutralization assay | Wild-type strain collection (No.: IVCAS 6.7512) | Vero E6 cells | Each serum  sample was first incubated at 56 °C for 30 min | The neutralizing titer was set as the dilution number of the 50% protective condition | Live SARS-CoV-2 neutralization assay | 38 convalescent human serum  samples were obtained from donors (18 to 83 years), with 16 (42.1%) mild cases, 19 (50%) moderate,1 (2.6%) severe case and 2 (5.3%) critical cases. | BetaCoV/Wuhan/AMMS01/2020 (EPI_ISL_5402124) | Vero E6 cells | Inactivated serum  was serially diluted 3-fold, starting at 1:5 | The neutralizing titer was set as the dilution number of the 50% protective condition | 1:5 (two were below the lower limit) | 53.6 | - |

# *In vitro* cross neutralization titer against both prototype and Delta variant

We also conducted a systematic search to update a previously-reported meta-analysis of in vitro neutralization titers of individuals who have been vaccinated with prototype-strain-based vaccines against both SARS-CoV-2 prototype strains and variants. Fold-reduction of neutralizing antibodies of the Delta variant compared to prototype strains were calculated by different types of neutralization assays. For the CoronaVac vaccine that didn’t have fold-reduction against the Delta variant, we used the data for an India inactivated vaccine (BBV152) with the same neutralization assay instead.

For the two mRNA vaccines, BNT162b2 and mRNA-1273, they performed neutralization assays for both prototype strains and the Delta variation by different sampling periods in their official reports. We extracted the mean fold-change of neutralization titer against the Delta variant (compared to wild-type strain) and corresponding 95% confidence interval. Specifically, the fold-change against the Delta variant was unknown for mRNA-1273 for the period of 14-30 days post the second dose, but the estimates for the Beta and Gamma were reported. Furthermore, it had been widely accepted that the reduction of neutralization antibody level for the Delta variant was not as significant as the Beta, while more apparent than the Gamma variant. In order to reduce heterogeneity led by lab-to-lab variations, we used the fold-change of the Beta variant for this specific time period instead and additionally conducted a sensitivity analysis by using fold-reduction of the Gamma variant. The summary of fold change of neutralization antibody level of the Delta variant compared to prototype strains were listed in **Table S6.**

### Table S8. Fold change of neutralization antibody level of the Delta variant compared to prototype strains

| Vaccine | Type of neutralization assay | Fold change from original study | Note | Data source |
| --- | --- | --- | --- | --- |
| mRNA-1273 | Pseudovirus neutralization assay | 18+ yrs  Month 1 post dose 2: 14.2 (12.0-23.3)  Month 6-8 post dose 2: 6.6 (2.7-11.6)  Day 14 post dose 3: 3.6 (1.8-4.3) | The fold change of the Delta variant was unknown on Month 1 post dose 2, we used the fold change of Beta variant instead in main analysis to provide a conservative estimate and conducted a sensitivity analysis by using the fold change of Gamma variant. As there were several studies that had proved that the reductions of neutralizing antibody level for the Delta was not as significant as the Beta, but more significant that the Gamma. | Choi et al., Safety and immunogenicity of SARS-CoV-2 variant mRNA vaccine boosters in healthy adults: an interim analysis. Nature medicine.  Slide 29-30 from Moderna 2Q 2021 Earnings Call |
| BNT162b2 | Live virus neutralization assay | 18-55 yrs:  Month 1 post dose 2: 1.3 (1.0-1.7)  Month 8 post dose 2: 2.1(0.9-4.5)  Month 1 post dose 3: 1.2 (0.6-2.4)  65-85 yrs:  Month 1 post dose 2: 1.5 (0.9-3.0)  Month 8 post dose 2: 2.1 (1.1-2.7)  Month 1 post dose 3: 1.1 (0.6-2.3)  18-85 yrs:  Month 1 post dose 2: 1.8 (1.3-2.5)  Month 8 post dose 2: 2.0 (1.3-3.1)  Month 1 post dose 3: 1.6 (1.1-2.5) | The fold change of the Delta variant was unknown on Month 8 post dose 2, we used the fold change of Beta variant instead and provided a conservative estimate. | Falsey et al., SARS-CoV-2 Neutralization with BNT162b2 Vaccine Dose 3, NEJM;  Slide 26-27 from Second Quarter 2021 Earnings Teleconference |
| CoronaVac | Live virus neutralization assay | 2.4 (1.1-5.2) | We assumed that the fold reduction for CoronaVac vaccine against the Delta variant was similar to an India inactivated vaccine (BBV152), and the fold change remained consistent overtime. | Chen X et al., Neutralizing antibodies against SARS-CoV-2 variants induced by natural infection or vaccination: a systematic review and pooled meta-analysis. Clinical Infectious Diseases 2021. |

# Predicting vaccine efficacy over time across different vaccines, variants and clinical endpoints

## 1. Predicting efficacy over time across different vaccines and clinical endpoints for other VOCs

### Figure S8. Predicting efficacy over time across different vaccines and clinical endpoints for other VOCs

(A) SARS-CoV-2 infection; (B) Symptomatic COVID-19; (C) Severe COVID-19

## 2. Sensitivity analysis predicted efficacy by changing fold-change parameter for mRNA-1273

Specifically, the fold-change against the Delta variant was unknown for mRNA-1273 for the period of 14-30 days post the second dose, and we conducted a sensitivity analysis by using fold-reduction of the Gamma variant. The predicted efficacy was 79.6% (95%CI: 67.5-89.2%) for mRNA-1273 between 14-30 days post dose 2, compared to the estimate of 63.5% (95%CI: 51.4-67.3%) in main analysis (using the fold-change of the Beta variant).

### Figure S9. Sensitivity analysis of predicted efficacy changing fold-change parameter for mRNA-1273

(A) SARS-CoV-2 infection; (B) Symptomatic COVID-19; (C) Severe COVID-19.

## 3. Sensitivity analysis of predicted efficacy by using confidence interval of model parameter slope (*k*)

### Figure S10. Sensitivity analysis of predicted efficacy by using upper limit of model parameter slope (k)

(A) SARS-CoV-2 infection; (B) Symptomatic COVID-19; (C) Severe COVID-19.

### Figure S11. Sensitivity analysis of predicted efficacy by using lower limit of model parameter slope (k)
